# Onychomadesis in a 20-Month-Old Child with Kawasaki Disease

**DOI:** 10.1155/2019/3156736

**Published:** 2019-03-03

**Authors:** Alexander K. C. Leung, Kin Fon Leong, Joseph M. Lam

**Affiliations:** ^1^Clinical Professor of Pediatrics at The University of Calgary, Calgary, Alberta, Canada T2M 0H5; ^2^Pediatric Consultant at The Alberta Children's Hospital, Calgary, Alberta, Canada T2M 0H5; ^3^Consultant Pediatric Dermatologist at the Pediatric Institute, Kuala Lumpur General Hospital, Kuala Lumpur, Malaysia; ^4^Clinical Associate Professor of Pediatrics and Associate Member at the Department of Dermatology and Skin Sciences, University of British Columbia, Vancouver, Canada

## Abstract

Kawasaki disease is characterized by fever for ≥ five days, bilateral bulbar conjunctival injection without exudate, polymorphous rash changes in the extremities, oral mucosal changes, and cervical lymphadenopathy. We report a 20-month-old boy with Kawasaki disease who had onychomadesis affecting the fingernails and toenails bilaterally. To our knowledge, there were three reported cases of onychomadesis associated with Kawasaki disease, to which we add another one. We suggest keeping in mind the possibility of onychomadesis as a nail sequela of Kawasaki disease.

## 1. Introduction

Kawasaki disease, also known as Kawasaki syndrome, is one of the most common acute systemic vasculitides that predominantly affects medium-sized arteries, with a predilection for coronary arteries [1]. The condition occurs mostly in children younger than five years of age [1]. The disease is characterized by fever of at least five days and a constellation of clinical features that are used as diagnostic criteria [2]. Onychomadesis has rarely been reported following Kawasaki disease [3–5]. A perusal of the literature revealed three cases, to which we are going to add another one.

## 2. Case Report

A 20-month-old Chinese boy was seen with a 7-day history of high-spiking fevers. The child broke out with a nonpruritic widespread reddish rash 1 day after the onset of fever. On the third day of the fever, he developed nonpurulent conjunctival injection. The child was irritable and had decreased oral intake. His mother brought him to see a family physician who treated the child with azithromycin and acetaminophen. The fever persisted in spite of the treatment. The child had not been exposed to anyone with a known infectious disease. His past medical history was unremarkable. The family history was noncontributory.

**Figure 1:**
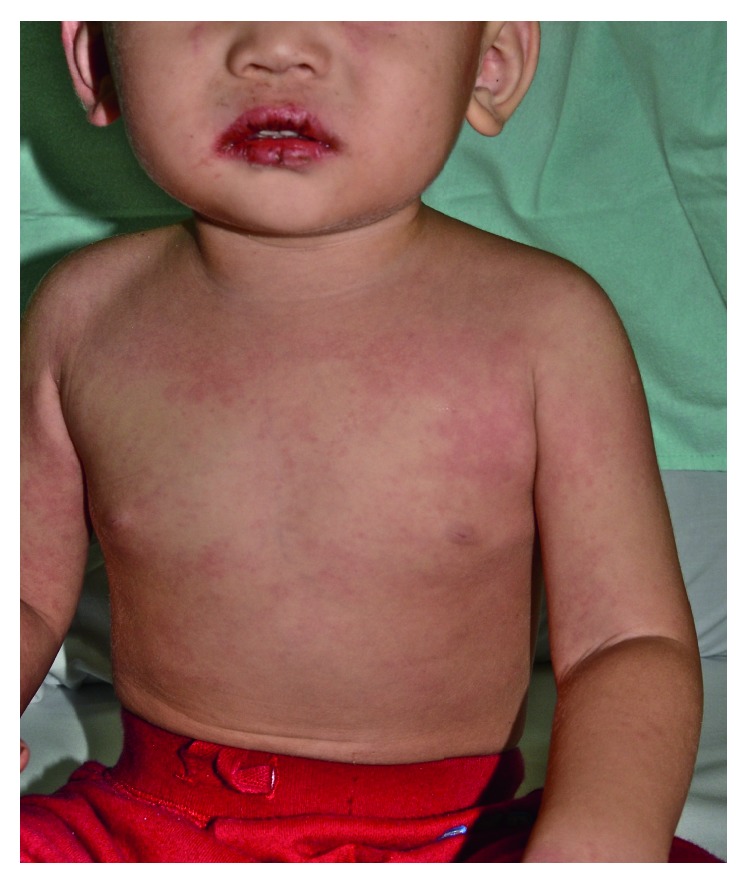
A 20-month-old boy with Kawasaki disease presenting with red cracked lips and an erythematous maculopapular rash on the anterior chest and upper arms.

On examination, the child was irritable and lethargic. His weight was 10.4 kg, height 82 cm, and head circumference 48.5 cm. His temperature was 39°C, heart rate 115 beats per minute, blood pressure 84/40 mm·Hg, and respiratory rate 33 breaths per minute. The child was noted to have bilateral nonpurulent bulbar conjunctival injection; fissured red lips (Figure 1); strawberry tongue diffuse erythema of the oropharyngeal mucosa; a generalized blanching polymorphous maculopapular rash over his face, trunk (Figure 1), and groin; erythema and firm edema of the dorsa of the hands and feet with sharp demarcation at the ankles and wrists and two enlarged firm tender lymph nodes each measuring 2 × 3 cm in the right cervical area. The rest of the physical examination was normal. In particular, there was no hepatosplenomegaly or a heart murmur.

The child was admitted to the hospital for investigations and management. Laboratory tests on admission revealed the following results: hemoglobin 12.6 g/dL (126 g/L), white blood cell count 21.3/*μ*L (×10^9^/L) with 88% neutrophils, platelet count 277 × 10^3^/*μ*L (×10^9^/L), and C-reactive protein 21.2 mg/L (201.7 nmol/L). Urinalysis showed 15 white blood cells per high-power field with no bacteria. Serum electrolytes, albumin, liver enzymes, and renal function were normal. Urine culture and throat swab culture were negative. The baseline chest radiograph, electrocardiograph, and echocardiograph were normal.

**Figure 2:**
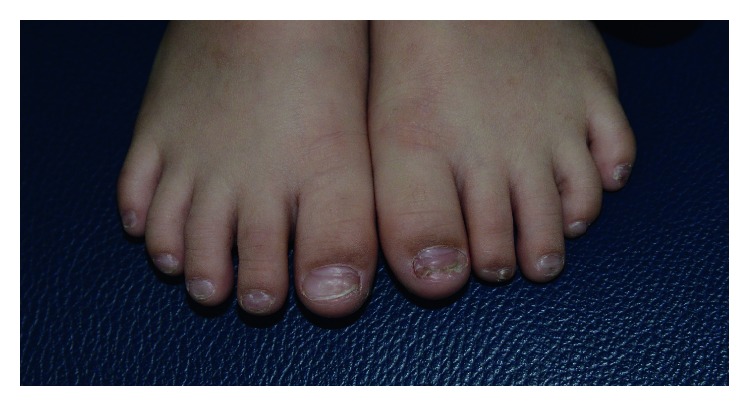
Onychomadesis noted at 2-month follow-up. The toenails on both feet were partially shed.

A diagnosis of Kawasaki disease was made based on the findings of fever for seven days, conjunctival injection, polymorphous rash, oral mucosal changes, changes in extremities, and cervical lymphadenopathy. The child was treated with intravenous immunoglobulin (2 g/kg) infused over 12 hours and high-dose aspirin (80 mg/kg/day divided into 4 doses) given orally. Over the next 36 hours, the child became afebrile, and the maculopapular rash resolved completely. He was discharged after 4 days of hospitalization on high-dose aspirin (80 mg/kg/day divided into 4 doses) for a total of 14 days followed by low-dose aspirin (4 mg/kg/day) in once-daily dosing for 8 weeks. At 2-month follow-up, the child's fingernails and toenails were found to be partially shed on both hands and feet, with the proximal nail beds covered by new nail (Figure 2). At 4-month follow-up, the old fingernails and toenails were fully shed, and the new fingernails and toenails were normal. Follow-up echocardiogram at 4 weeks and 3 months was normal.

## 3. Discussion

The diagnosis of classic or typical Kawasaki disease is based on clinical criteria established by the American Heart Association [2]. These criteria include fever for ≥ five days (first calendar day of temperature is illness day 1) plus ≥ four of the five primary or principal clinical features without plausible alternative explanations for the clinical findings. The five principal clinical features are bilateral bulbar conjunctival injection without exudate, polymorphous rash (diffuse maculopapular, erythroderma, urticarial, erythema multiforme-like, but not bullous or vesicular), changes in the extremities (periungual desquamation, indurated edema and erythema of the feet and hands, and sharp demarcation at the ankles and wrists), oral mucosal changes (erythematous, fissured cracked lips, diffuse erythema of the oral pharynx, and strawberry tongue), and cervical lymphadenopathy (unilateral, nonfluctuant, >1.5 cm in diameter) [2]. According to the 2017 scientific statement for health professions from the American Heart Association, in the presence of ≥4 major features, mainly when swelling and redness of the feet and hands are present, the diagnosis can also be made even if the fever has only been present for four days [2]. In this regard, the Japanese criteria include those cases in whom the fever has subsided before the fifth day in response to treatment [6]. Patients who have a fever for five or more days and only three major clinical features can also be diagnosed as having classic Kawasaki disease when coronary artery disease is detected by two-dimensional (2D) echocardiography or coronary angiography [2].

Periungual desquamation of the fingers and toes is a characteristic feature of Kawasaki disease and usually occurs 2 to 3 weeks after the onset of fever [1]. Nail changes in Kawasaki disease include Beau lines (linear nail creases), transverse orange-brown or red chromonychia, transverse leukonychia (leukonychia striata), pincer nails, and onychomadesis, usually occurring four to six weeks after onset of fever [7–11].

Onychomadesis refers to the separation of the nail plate from the matrix starting at the proximal edge and is the result of temporary arrest of growth of the nail bed matrix [12]. The mechanism of nail matrix arrest is unknown but may be secondary to inhibition of cellular proliferation [13]. It is also possible that the quality of the newly formed nail may be temporarily altered, leading to separating of the nail plate [13]. As the new nail begins its growth proximally, it extends underneath the previous nail, pushing the previous nail forward, resulting in shedding of the previous nail [13, 14]. Typically, the condition is painless and self-limited [14]. New nail growth will be normal [14]. Onychomadesis may occur in fingernails and/or toenails. Although onychomadesis is usually easily recognizable, it can be mistaken for onychomycosis, trachyonychia, or psoriatic nails. Onychomadesis has been shown to be associated with infections (notably hand-foot-mouth disease, varicella, scarlet fever, onychomycosis, and paronychia), systemic medical illnesses (Kawasaki disease, Steven-Johnson syndrome, toxic epidermal necrolysis, lichen planus, Cronkhite–Canada syndrome, Guillain-Barré syndrome, myocardial infarction, renal failure, and immunodeficiency), direct trauma to the nail matrix, medications (chemotherapeutic agents [doxorubicin, capecitabine, etoposide, and cytosine arabinoside], antibiotics [penicillin, cloxacillin, azithromycin, and cephalosporin], antiepileptics [valproic acid and carbamazepine], retinoids, lithium, and lead), and autoimmune diseases (alopecia areata and pemphigus vulgaris) [5,12–14]. Onychomadesis can also be familial or idiopathic [13].

Onychomadesis associated with Kawasaki disease is very rare. To our knowledge, only three cases have been reported in the literature [3–5]. In 1990, Pilapil and Quizon reported a 4-year-old Caucasian boy with features of Kawasaki disease for 7 days before he was hospitalized and treated as such for 20 days [5]. The patient was noted to have onychomadesis affecting his fingernails and toenails when he was examined 36 days after discharge from the hospital. In 2002, Ciastko described an 8-year-old boy with Kawasaki disease who was noted to have onychomadesis of the fingernails and toenails 6 weeks from the onset of the illness [3]. In 2015, Kalasekhar and Venkatesh reported a 2-year-old boy with Kawasaki disease [4]. On the 10th day of the illness, the child was noted to have orange-brown discoloration on the right ring fingernail, transverse nail crease on his right middle fingernail, and onychomadesis of the right index fingernail [4]. We report a 20-month-old child with Kawasaki disease who had onychomadesis on the fingernails and toenails bilaterally two months from the onset of the disease.

Our impression is that onychomadesis associated with Kawasaki disease may be more common than is generally appreciated. We suggest keeping in mind the possibility of onychomadesis as a nail sequela of Kawasaki disease.
